# In Vitro Inhibition of Enzymes and Antioxidant and Chemical Fingerprinting Characteristics of *Azara serrata* Ruiz & Pav. Fruits, an Endemic Plant of the Valdivian Forest of Chile

**DOI:** 10.3390/plants13192756

**Published:** 2024-09-30

**Authors:** Philipp Hopfstock, Javier Romero-Parra, Peter Winterhalter, Recep Gök, Mario Simirgiotis

**Affiliations:** 1 Institute of Food Chemistry, Technische Universität Braunschweig, Schleinitzstraße 20, 38106 Braunschweig, Germany; p.hopfstock@tu-braunschweig.de (P.H.); p.winterhalter@tu-braunschweig.de (P.W.); 2 Departamento de Química Orgánica y Fisicoquímica, Facultad de Ciencias Químicas y Farmacéuticas, Universidad de Chile, Santiago 6640022, Chile; javier.romero@ciq.uchile.cl; 3 Instituto de Farmacia, Facultad de Ciencias, Universidad Austral de Chile, Valdivia 5110566, Chile

**Keywords:** corcolen, salicaceae, endemic berries, enzyme inhibition, antioxidants, anthocyanins, pyrano anthocyanins, analytical membrane chromatography, collision cross-section, TIMS-TOF

## Abstract

The World Health Organization has emphasized the importance of consuming small fruits for the prevention of chronic health problems, including diabetes, cardiovascular diseases, cancer, and obesity, which are named chronic non-communicable diseases (NCDs). *Azara serrata* Ruiz & Pav., commonly called “aroma de Castilla”, is a shrub endemic to Chile from the Salicaceae family that produces an underutilized blue-grey berry that grows wild in southern Chile. The species is widely used as a medicinal plant by the Andean communities of southern Chile. In this work, a high-resolution mass spectrometric analysis of the methanolic extract revealed several phenolic compounds for the first time in the edible berry of this endemic species. Furthermore, several glycosylated anthocyanins were detected and quantified using UHPLC coupled with UV/Vis detection and trapped ion mobility mass spectrometry (UHPLC-DAD-TIMS-TOF) for the anthocyanin-rich extract, which was prepared using an optimized anthocyanin extraction protocol. The extract proved to be active in the inhibition of several enzymes linked to NCDs, such as acetylcholinesterase, tyrosinase, amylase, lipase, and glucosidase (IC_50_ = 3.92 ± 0.23, 12.24 ± 0.03, 11.12 ± 0.10, 32.43 ± 0.0, and 371.6 ± 0.0 μg/mL, respectively). Furthermore, the extract concentrated in anthocyanins showed good antioxidant activity evidenced by the bleaching of the radicals DPPH and ABTS, ferric-reducing antioxidant power (FRAP), and oxygen radical absorbance capacity (ORAC). The results show that these neglected endemic small berries can be a source of healthy phytochemicals. These Chilean berries can be used as functional food and their extracts are candidates for use as functional ingredients in naturally healthy products.

## 1. Introduction

**Figure 1:**
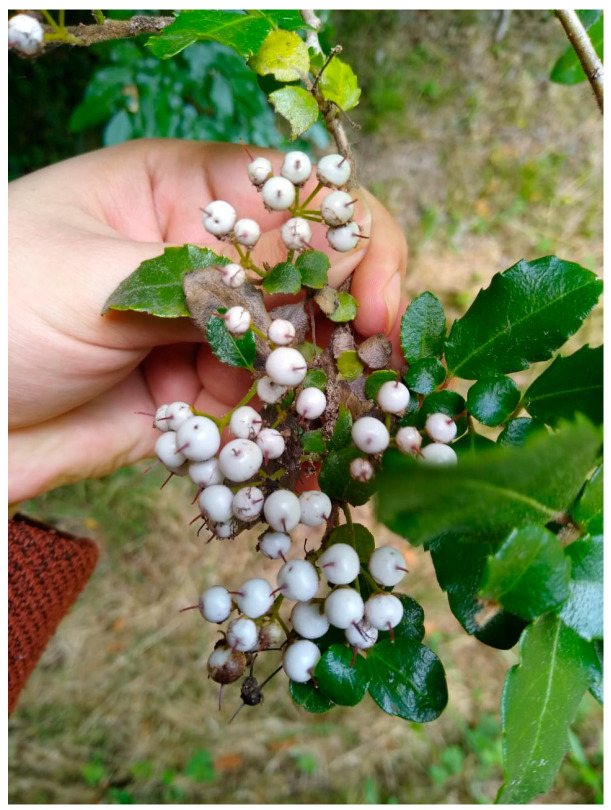
*Azara serrata* Ruiz & Pav. fruits and its leaves, collected in February 2022 from the Botanical Garden, UACh, Valdivia.

The Valdivian Forest has diverse endemic species with interesting compounds that are at risk of extinction including edible small berries. Several phenolic compounds and glyco-sylated anthocyanins for some representative endemic species berries have been reported previously [1,2]. With ten species of flowering plants belonging to the Salicaceae family, the genus *Azara* is native to temperate to subtropical parts of South America [3]. They are primarily found in Chile from the towns of Coquimbo to Valdivia, usually along lakeshores and the edges of forests. The leaves of the shrubs and small trees are alternating; however, in some species, they appear paired, measuring 1–4 cm long by 0.5–5 cm broad. The plants can reach heights of 1–8 m. They have black to red berries that are 3–10 mm broad. The plants are common in Chile, but there are not many research articles from Chile on this genus. A new myricetin 3-*O*-α-l-dirhamnoside was reported from *Azara microphylla* (local name corcolén) a long time ago [4]. Subsequently, a variety of phenolic compounds, including caffeic and coumaric acids, and three flavonoids (pinocembrin, chrysin, and luteolin) have been reported recently in honey and propolis made of *A. petiolaris* and *A. integrifolia* [5]. For *Azara dentata* Ruiz & Pav., local name “corcolen blanco”, cytotoxic effects and the presence of coumarins, sterols, and other components in the methanolic extract of leaves by GC-MS have been reported [6]. More recently, Ramos et al. (2023) also reported the antioxidant activity, enzymatic inhibition, and relaxation effects in rat aorta induced by the ethanolic extract of these fruits [7]. These neglected and understudied endemic Chilean species can offer novel substances or bioactivities that merit further study, especially the antifeedant compounds known as salicin glycoside derivatives [8]. However, due to the large structural variability of compounds in the Salicaceae family, which is mainly due to polar phenolic compounds, and the high number of different structures discovered so far, a thorough analysis and separation of phenolic compounds in their edible fruits is necessary [9]. *Azara serrata* Ruiz & Pav. (local name Aromo de Castilla) produces a white-blue-grey berry (Figure 1). In Chile, this plant grows in the regions of Coquimbo, Valparaíso, Santiago, O’Higgins, Maule, Biobío, Araucanía, and Los Ríos and is reported in the ancient literature to be used in Mapuche medicine as an antirheumatic and antitussive [3].

Chronic or noncommunicable diseases (NCDs) are long-lasting conditions that often progress slowly. The primary forms are diabetes, cancer, chronic respiratory diseases (such as asthma and chronic obstructive lung disease), and cardiovascular diseases (such as heart attacks and stroke) (WHO) [10]. Together with neurodegenerative diseases (Alzheimer’s, Parkinson’s, and others; Alzheimer’s accounts for roughly 70% of dementia cases), these illnesses are the primary causes of premature deaths globally [10]. Berries are the primary source of phenolic compounds in diets, and health-promoting phenolics have been found to be present in several South American berries [11,12,13,14]. Indeed, berries and native fruits from unique habitats, like the Chilean terroir, can yield intriguing and complex phytochemicals that might serve as models for exploring potential new uses for them as food additives, nutraceuticals, or herbal remedies. In this study, through UHPLC-DAD-TIMS-TOF analyses, we offer a variety of novel approaches to the chemical and biological activity of underappreciated *Azara serrata* fruits regarding antioxidant properties and the inhibition of enzymes related to NCDs.

Anthocyanins, as secondary plant metabolites, prominently occur in berries, vegetables, and fruits of a large variety of plant families [15]. As for the plant family of salicaceae, it has been reported that anthocyanins can occur in the bark of *Salix purpurea* and some of its hybrids [16]. Another representative of the anthocyanins found in the Salicaceae plant family is said to be various species of *Populus* L. [17]. The species *Populus nigra* L. is reported to contain anthocyanins such as cyanidin, delphinidin, petunidin, and pelargonidin [17]. Only a limited number of salicaceae plants produce fruits such as *Azara serrata* Ruits & Pav. The plant *Idesia polycarpa* produces orange-red berries, which are likely to contain carotenoids due to their color and high oil content, which has been reported to be up to 26.26% in seeds and 43.6% in the pulp (dry weight) [18]. Other investigated salicaceaes that produce fruits containing anthocyanins are *Dovyalis hebecarpa* native to Sri Lanka and southern India and *Flacourtia jangomas*, which is cultivated in the southeast of Asia [19,20].

A large-scale review has pointed out that anthocyanins show positive effects on cardiovascular health and vision. They also possess antidiabetic, neuroprotective, antimicrobial, and anti-inflammatory properties and are related to chemopreventive effects against cancer [21]. The anthocyanins in red raspberries, mainly containing different glycosides of cyanidin and some pelargonidins, for example, have been shown to modulate proteins involved in antioxidant, anticancer, and anti-inflammatory effects in cultured cells [22]. However, the authors of the review emphasized that in vitro models cannot be directly translated to in vivo studies without concerns, especially regarding the concentration levels used in in vitro studies compared to bioavailability and metabolism in vivo. Therefore, it is crucial to maintain a critical perspective regarding the potential health benefits of anthocyanin-rich foods [22].

In this study, we present the results of an analysis of berries from *Azara serrata* Ruiz & Pav. by UHPLC-DAD-TIMS-TOF, as well as results from different assays regarding the content of various substance classes. Additionally, docking studies were performed for a selection of compounds occurring in the berries.

## 2. Results and Discussion

### 2.1. Phenolic Profile of Azara serrata Ruiz & Pav.

**Figure 2:**
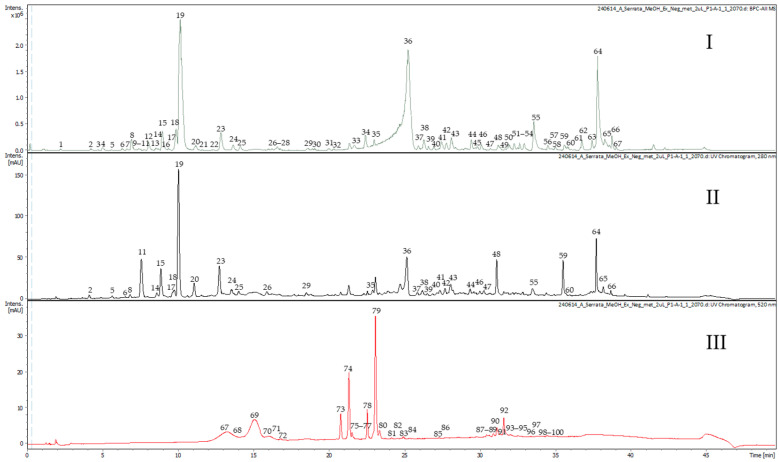
UHPLC-DAD-TIMS-TOF-MS analysis of the methanolic extract of the berries from *Azara serrata* Ruiz & Pav., with base peak all MS ne—gative (**I**), DAD signal at 280 nm (**II**), and DAD signal at 520 nm (**III**).

The base peak chromatograms (ESI-negative) as well as UV/Vis at wavelengths of 280 nm and 520 nm of a methanolic extract of the dried berries from *Azara serrata* Ruiz & Pav. are illustrated in Figure 2. The negative system operation mode was used for the elucidation of the methanolic extract and the positive mode was used for the analysis of the anthocyanins. Table 1 shows the results of the spectrometric investigation by UHPLC-DAD-TIMS-TOF analysis, which includes high-resolution mass spectral data and fragmentation and ion mobility data in the form of CCS values. The compounds were tentatively annotated. The UHPLC-DAD-TIMS-TOF-MS analysis of the purified anthocyanin extract (cf. Section 3.3.3) in positive operation mode is in the Supplementary Materials Figure S1. A detailed explanation is below.

#### 2.1.1. Phenolic Acids

**Figure 3:**
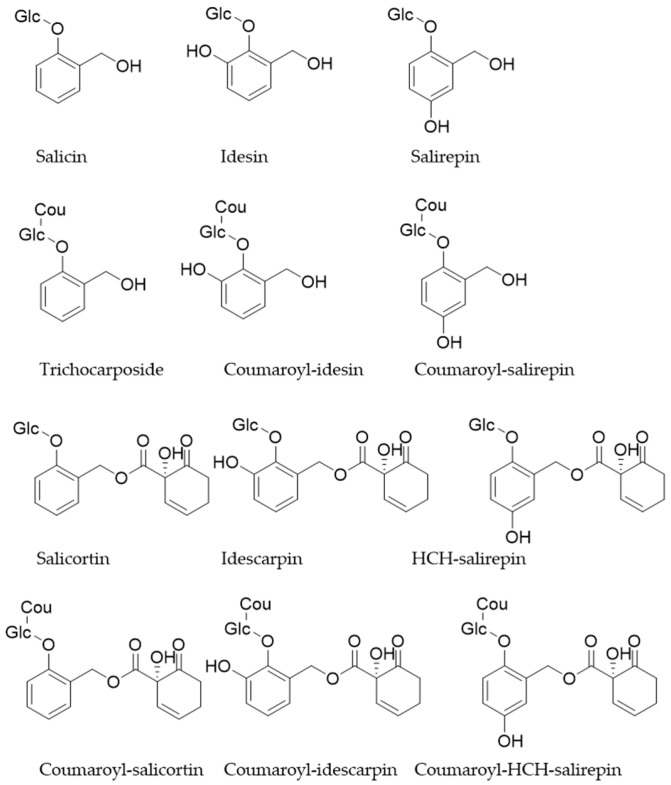
Chemical structures of some salicinoids in the berries of *Azara serrata* Ruiz & Pav.

According to our data, the extract contained various species of quinic acid derivates like chlorogenic acid isomers, [M − H]^−^ theoretical *m/z* 353.0878 (**15**, **23**, **25**), and feruloyl quinic acid isomers, [M − H]^−^ theoretical *m/z* 367.1035 (**24**, **29**). Most of the signals could be assigned to phenolic glycosides, which have been reported to occur in the plant family of Salicaceae [9,23]. The main phenolic glycosides observed were annotated as idesin or its isomer salirepin, [M − H]^−^ theoretical *m/z* 301.0928 (**8**, **11**, **19**), which both have been isolated from the Salicaceae *Idesia polycarpa* Maxim., native to the eastern part of Asia [24]. Further, it is described that in the presence of 1-hydroxy-6-oxo-2-cyclohexene-1-carboxylic acid (HCC), salicin as well as idesin form the compounds salicortin, [M − H]^−^ theoretical *m*/*z* 423.1657 (**43**, **44**, **47**), and idescarpin or its salirepin analog, [M − H]^−^ theoretical *m/z* 439.1246 (**33**, **36**, **48**, **59**) [25,26]. Salicortin as well as some of its structural relatives have been isolated from the bark of willow trees (*Salix* spp.) [26]. Both idescarpin and salicortin have shown promising pharmaceutical benefits in different studies [9]. Further coumaroyl derivates of those species have been annotated. The coumaroyl species of salicin are trichocarposide, [M − H]^−^ theoretical *m/z* 431.1348 (**37**, **41**, **46**, **51**, **53**); coumaroyl idesin, [M − H]^−^ theoretical *m/z* 447.1297 (**50**); and coumaroyl idescarpin, [M − H]^−^ theoretical *m/z* 585.1614 (**63**). As described in the literature, the hexose moiety of salicinoids can be acetylated as well, resulting in more structural variety [26]. Like the coumaroylated species, three different isomers of an acetylated coumaroyl idescarpin or the salirepin analoga have been detected, [M − H]^−^ theoretical *m/z* 627.1719 (**64**, **65**). Figure 3 illustrates the major salicinoids present in the methanolic extract of the berries of *Azara serrata* Ruiz & Pav., as reflected in their detector counts (MS) and UV/Vis absorbance (cf. Figure 2).

#### 2.1.2. Anthocyanins

**Figure 4:**
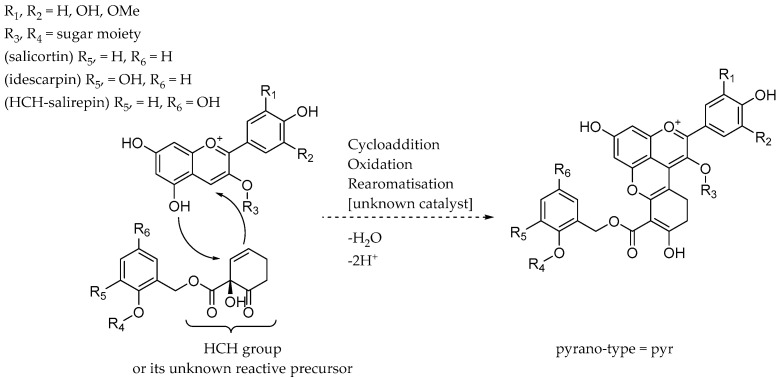
Proposed mechanism of the formation of the pyranoanthocyanin species in the berries of *Azara serrata* Ruiz & Pav.

Apart from the more commonly occurring cyanidin- and delphinidin-glycosides (**67**–**73**), which have also been reported by Bridle Stott et al. (1973) in *Salix purpurea*, most of the observed anthocyanins have not been, to the best of our knowledge, described yet and therefore represent potential novelties [16,27]. We postulate that these previously unreported types of pyranoanthocyanin are formed by a reaction of the respective anthocyanin with the double bond within the HCH unit of idescarpin or the salirepin isomer, as illustrated in Figure 4. The reaction may involve an unknown precursor of the HCH molecule, as well as a catalyst, which could be an enzyme or external stimulus.

The chromatographic pattern, as well as the mass difference of *m/z* 16 between delphinidin and cyanidin, persisted throughout the analysis, but the typical fragments of *m/z* 287 for cyanidin and *m/z* 303 for delphinidin could not be detected. Apparently, all of the novel anthocyanins (**74**–**100**) have a fragment of *m/z* 377 for the cyanidin species and *m/z* 393 for the delphinidin species. An important observation throughout the analysis was the difference in the CCS values between the cyanidin and delphinidin species, being ∆ 3.7 Å^2^ for the glucosides, [M]^+^ *m/z* 465.1023 and [M]^+^ *m/z* 449.1075 (**67** & **69**), and ∆ 3.3 Å^2^ for the rutinosides, [M]^+^ *m/z* 611.1608 and [M]^+^ *m/z* 595.1660 (**68** & **70**), which remained in the same order of magnitude. Therefore, we observed ∆ 3.5 Å^2^ for the glucosides, [M]^+^ *m/z* 885.2084 and [M]^+^ *m/z* 869.2135 (**75** & **79**), of the other species and ∆ 2.5 Å^2^ for the rutinosides, [M]^+^ *m/z* 1031.2664 and [M]^+^ *m/z* 1015.2173 (**74** & **78**). This observation carried through to the higher mass anthocyanins eluting at the end of the chromatographic program (**85**–**100**), which were the coumaroyl and acetylated counterparts of the idesin, respectively, salirepin and analogas, described above. Because of this very similar pattern regarding the factors—retention time, the difference in the mass-to-charge ratio of 16, and the almost identical difference in the CCS values between cyanidin and delphinidin species—we are confident in our observation that the here-found anthocyanin species originate from the cyanidin- and delphinidin-glycosides present in the plant. Additionally, we observed a shift in the UV/Vis maximum towards 500 nm for these substances, which has been described as characteristic of pyranoanthocyanins [28,29]. This subclass of the anthocyanins is often reported in red wines, resulting from a condensation reaction between malvidin-glycosides and other phenolic compounds found in red wines [27]. A wide range of pyranoanthocyanins in red wine has been described. The group known as vitisins is formed by a reaction between pyruvic acid, acetaldehyde, acetoacetic acid, or acetone with malvidin-glycosides. Another group referred to as pinotins is formed by the reaction of anthocyanins with a vinyl compound such as different vinylphenols or hydroxycinnamic acids [30]. Pyranoanthocyanin-flavanols are formed by the reaction of 8-vinylcatechin with the respective anthocyanin [27]. Lastly, the group of vinylpyranoanthocyanins, termed portisins, is described in red wines due to the reaction of the already-formed carboxypyranoanthocyanin with either vinylflavan-3-ol or hydroxycinnamic acid [31]. Apart from red wines, pyranoanthocyanins have been isolated from blood orange juice (*Citrus sinensis* L.) [32], black carrot juice (*Daucus carota* L.) [33], strawberry (*Fragaria ananassa*) [34], red onion (*Allium cepa*) [35], and, recently, wines made from bilberry (*Vaccinium myrtillus* L.) [36].

### 2.2. Quantitation of Anthocyanins

**Figure 5:**
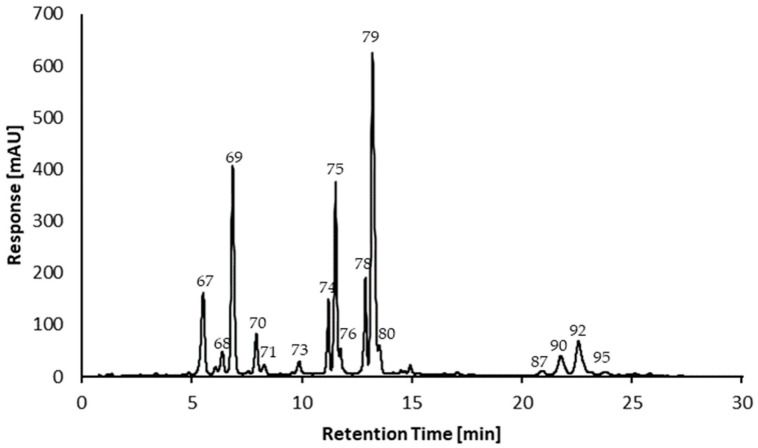
Chromatogram of the UHPLC-DAD analysis of the anthocyanin-rich extract of the berries of *Azara serrata* Ruiz & Pav. at 520 nm.

A chromatogram of the UHPLC-DAD analysis of the enriched anthocyanin extract is depicted in Figure 5. Preparation of the extract using analytical membrane chromatography (cf. Section 3.3.3) was carried out in triplicate; for comparison, a chromatogram of the purified extract at the wavelength of 280 nm is in the Supplementary Materials (Figure S2).

For quantitation purposes, only anthocyanins with an S/N > 10 were considered. Therefore, the content of the major anthocyanins is summarized in Table 2. Quantitation was performed using a cyanidin-3-*O*-β-d-glucoside calibration curve ranging from 1 to 75 mg L^−1^; the curve including its key figures is in the Supplementary Materials Figure S3.

The most abundant anthocyanin was cyanidin-3-*O*-β-d-glucoside, with a content of 115.8 ± 9.38 µg g^−1^ (DW), followed by delphinidin-3-*O*-β-d-glucoside with 81.20 ± 8.00 µg g^−1^ (DW). For the pyranoanthocyanin species, a similar observation was made, with the pyranocyanidin-type glucoside having a content of 75.55 ± 4.67 µg g^−1^ (DW) and the pyranodelphinidin-type glucoside having a content of 37.73 ± 3.13 µg g^−1^ (DW). Since the contents are expressed as cyanidin-3-*O*-β-d-glucoside equivalents, we assume that the actual content of the pyranoanthocyanins might have been slightly higher due to the shift in the maximum light absorbance. The total anthocyanin content in the dry berries of *Azara serrata* Ruiz & Pav. was 441.49 ± 34.93 µg g^−1^ (DW). Given that the dry weight was approximately 1/10th of the fresh weight, the anthocyanin content was in the same order of magnitude as that of red currant (*Ribes* L.), whose berries have been reported to contain 4.95 ± 0.24 mg 100 g^−1^ of anthocyanins at fresh weight [37]. With 60.26% of the total anthocyanin content, the cyanidin species was more abundant in the plant compared to the 39.74% total content of the delphinidin species.

### 2.3. Phytochemical and Antioxidant Activity

#### Comparative Determination of Total Anthocyanins, Phenols, Flavonoids, and Antioxidant Capacities

Phenolic compounds, known for their strong antioxidant properties, are secondary metabolites found in various plant species. The total phenolic and flavonoid contents of the fruits are depicted in Table 3. The values for TAC were several times less than those reported for the Chilean maqui berries *Aristotelia chilensis* (between 22 and 50 mg/g DW) [11,38]; however, TPC was higher than that of blueberries Bluegold, Brigitta, and Draper (19.12, 10.54, and 15.69 mg GAE/g DW, respectively); murta berries (34.92 mg GAE/g DW); and maqui berries (31.16 mg GAE/g DW) [39]. These differences may be due to the different and strong phenolic compounds detected in this plant belonging to salicaceae. On the other hand, ORAC values were also close to those of blueberries Bluegold, Brigitta, and Draper (336, 213, and 333 μmol TE/g dry weight, respectively) and maqui berries (371 μmol TE/g dry weight) and higher than those of murta *Ugni molinae* berries (222.78 μmol TE/g dry weight) [39]. TFC was also higher than that reported for arrayan *Luma apiculata* and *Ugni molinae* fruits (29.44 and 5.54 mg QE/g dry weight, respectively) [1]. Bleaching of ABTS radicals was more sensitive than DPPH, while ferric-reducing antioxidant power (FRAP) showed the same trend as ORAC (Table 3).

### 2.4. Enzyme Inhibitory Properties of Azara serrata. Ruiz & Pav.

Several important enzymes related to NCD were assayed with the methanolic extract from the *A. serrata* endemic fruits. The results are shown in Table 4. Some of the enzymes involved in the development of metabolic syndromes are glucosidases, amylases, and lipases. Starches are the main foods in the human diet and several enzymes participate in the digestion of carbohydrates, such as amylases in the saliva and pancreas, which act on oligosaccharides and fragment them into monosaccharides for their consequent absorption. Indeed, type-2 diabetes mellitus is one of the most prevalent metabolic diseases in the world and is characterized by hyperglycemia. Alpha-glucosidase and alpha-amylase are hydrolases that release glucose by digesting glycogen and starch and are involved in diabetes and a variety of other diseases, such as infections and cancer [40]. The inhibition of α-amylase and α-glucosidase retards the digestion and absorption of carbohydrates and subsequently suppresses postprandial hyperglycemia. The inhibition can benefit patients with altered glucose metabolism because it can retard carbohydrate absorption, producing a lowering effect on postprandial insulin levels and glucosa in the blood [41,42]. The search for natural inhibitors such as flavonoids and phenolic acids [42] could lead to the discovery of a useful natural-origin drug for subsequent treatment. In this study, the most remarkable results were of amylase (IC_50_: 7.23 ± 0.0), which was close to that of standard acarbose (IC_50_: 6.56 ± 0.0), even though glucosidase inhibition was low compared to acarbose and lipase inhibition was IC_50_: 32.43 ± 0.0, 15 times lower than the standard drug orlistat (Table 4). On the other side, cholinesterase enzymes (ChE) play a major role in the development of Alzheimer’s disease because they catalyze the hydrolysis and inactivation of the acetylcholine neurotransmitter, yielding choline and acetate. Cholinesterase inhibitors such as some phenolic compounds improve the cholinergic function of AD, preserving the levels of acetylcholine, and are therefore a good approach in the symptomatic treatment of AD [43]. The inhibition of these enzymes could be helpful also for dementia and Parkinson’s disease [44], autism, and schizophrenia [45]. In this research, other important inhibition results were of cholinesterases (*h*BuChE IC_50_: 12.24 ± 0.03 and *Tc*AChE IC_50_: 3.92 ± 0.23), which were not too distant from those of the standard inhibitor drug galantamine (*h*BuChE IC_50_: 3.81 ± 0.02 and *Tc*AChE IC_50_: 0.57 ± 0.03). Finally, tyrosinase inhibition of the *Azara* methanolic extract was 12 times lower than that of standard kojic acid (Table 4).

#### 2.4.1. Docking Calculations

**Figure 6:**
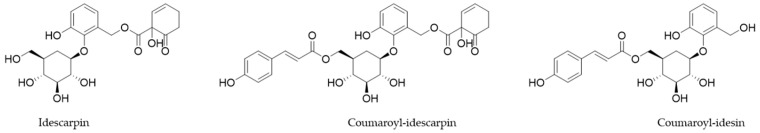
Structures of a selection of salicinoids in the berries of *A. serrata* used for the docking calculations.

Identified idescarpin, coumaroyl idescarpin, and coumaroyl idesin (Figure 6) from *A. serrata* methanolic extract as well as the corresponding enzymatic inhibitors (galantamine, kojic acid, acarbose, and orlistat) were subjected to docking assays into each enzyme catalytic site to analyze the docking energy descriptor and the different molecular interactions between the amino acids of the catalytic site and the already-mentioned chosen molecules. The latter aimed to provide a rationale for the inhibition activities observed over the enzymes by the extract (see Table 4).

##### Acetylcholinesterase (*Tc*AChE) Docking Results

**Figure 7:**
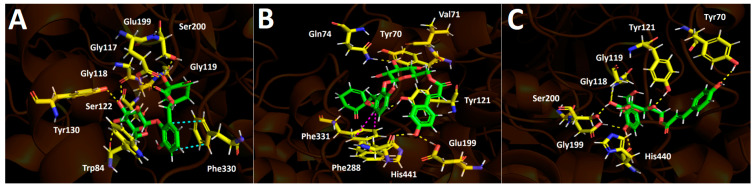
Predicted intermolecular interactions of selected compounds in *A. serrata* methanolic extract and the residues of the Torpedo Californica acetylcholinesterase (*Tc*AChE) catalytic site. Yellow dotted lines indicate hydrogen bond interactions, cyan dotted lines represent π-π interactions, and magenta dotted lines represent T-shaped interactions. (**A**). Idescarpin in the catalytic site. (**B**). Coumaroyl idescarpin in the catalytic site. (**C**). Coumaroyl idesin in the catalytic site.

Idescarpin, coumaroyl idescarpin, and coumaroyl idesin, listed in Table 5, mainly engaged in hydrogen bond interactions with the residues within the acetylcholinesterase catalytic site. Idescarpin formed several hydrogen bonds due to the nature of its structure, which possesses many oxygen atoms and polar hydrogen atoms. Therefore, idescarpin formed eight hydrogen bonds with Trp84, Gly117, Gly118, Gly119, Ser122, Tyr130, Glu199, and Ser200, as well as a π-π interaction with Phe330 (see Figure 7A). Coumaroyl idescarpin corresponds to the idescarpin molecule but esterifies with p-coumaric acid. Consequently, it shared the ability of the former to carry out multiple hydrogen bond interactions with the amino acids of the acetylcholinesterase catalytic site. The amino acids involved were Tyr70, Val71, Gln74, Tyr121, Glu199, Phe288, and His440 in hydrogen bond interactions, and there was a T-shaped interaction between Phe331 and the benzene ring of the idescarpide scaffold of coumaroyl idescarpide (Figure 7B). The latter indicated that both derivatives arranged in different manners into the catalytic site of the enzyme. On the other hand, coumaroyl idesin bears p-coumaric acid esterified with a sugar moiety and possesses a different hydroxymethylphenol ring compared to coumaroyl idescarpide. As can be seen in Figure 7C, this derivative performed seven hydrogen bond interactions with Tyr70, Tyr121, Gly118, Gly119, Ser200, Glu199, and His440. No π-π or T-shaped interactions were exhibited by the coumaroyl idesin.

##### Butyrylcholinesterase (*h*BuChE) Docking Results

**Figure 8:**
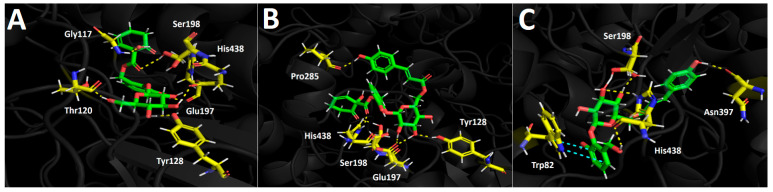
Predicted intermolecular interactions of selected compounds in *A. serrata* methanolic extract and the residues of the human butyrylcholinesterase (*h*BuChE) catalytic site. Yellow dotted lines indicate hydrogen bond interactions and cyan dotted lines represent π-π interactions. (**A**). Idescarpin in the catalytic site. (**B**). Coumaroyl idescarpin in the catalytic site. (**C**). Coumaroyl idesin in the catalytic site.

Compounds selected from the *Azara serrata* methanolic extract demonstrated favorable binding energies when subjected to docking experiments targeting the butyrylcholinesterase catalytic site. Once again, as seen in acetylcholinesterase docking descriptors, hydrogen bond interactions predominantly occurred with the residues of the catalytic pocket in all three derivatives tested. Idescarpin, which exhibited the best binding energy (−15.512 kcal/mol, see Table 5), formed six different hydrogen bond interactions with Gly117, Thr120, Tyr128, Glu197, Ser198, and His438 (Figure 8A). On the other hand, the binding energy exhibited by coumaroyl idescarpin was of the same order of magnitude as that of idescarpin (−15.115 kcal/mol). Although coumaroyl idescarpin formed one more hydrogen bond interaction than idescarpin (Figure 8B), the former also had greater contact with the water solvent due to the p-coumaric acid core in its structure. The solvent contact could explain the slightly unfavorable energy for this derivative, which formed hydrogen bonds with Tyr128, Glu197, Ser198, Pro285, and His438. Figure 8C shows the coumaroyl idesin arrangement in the butyrylcholinesterase catalytic site. This derivative formed four hydrogen bond interactions with Ser198, Asn 397, and His438, as well as one π-π interaction between Trp82 and the phenyl ring of the idesin moiety. Even though coumaroyl idesin formed less interactions compared with the other evaluated compounds, it also showed a good energy profile (−12.103 kcal/mol).

##### Tyrosinase Docking Results

Inhibition assays of the *A. Serrata* methanolic extract over tyrosinase showed similar potency to the known inhibitor kojic acid (11.12 ± 0.10 µg/mL and 0.73 ± 0.04 µg/mL, respectively, see Table 4). In this sense, the binding energies of the selected compounds that underwent docking assays were consistent with the experimental evidence of enzyme inhibition employing the inhibitor (Table 5). Therefore, the energy profiles suggested that each tested derivative could exert a similar inhibition potency over the tyrosinase enzyme.

**Figure 9:**
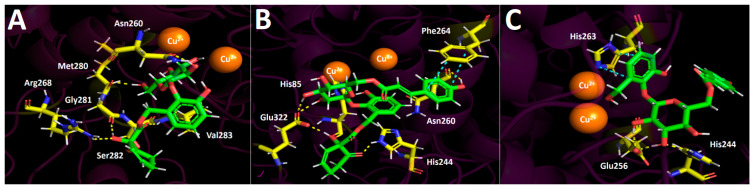
Predicted intermolecular interactions of selected compounds in the *A. serrata* methanolic extract and the residues of the human *Agaricus bisporus* mushroom tyrosinase catalytic site. Yellow dotted lines indicate hydrogen bond interactions and cyan dotted lines represent π-π interactions. (**A**). Idescarpin in the catalytic site. (**B**). Coumaroyl idescarpin in the catalytic site. (**C**). Coumaroyl idesin in the catalytic site.

Concerning the intermolecular interactions of the selected compounds, the docking descriptors suggested that mainly hydrogen bond interactions with the residues of the catalytic site occurred, except for coumaroyl idesin, which presented fewer of these interactions and consequently had the least favorable energy profile. Although it presented a π-π interaction with His263, this interaction did not appear to contribute significantly to the binding energy (Figure 9A). Idescarpin formed hydrogen bonds with Asn260, Arg268, Met280, Gly281, Ser282, and Val283, while coumaroyl idescarpin showed hydrogen bond interactions with His85, His244, Glu322, and Asn260 and one π-π interaction with Phe264 through the phenyl aromatic ring of p-coumaric acid core ester (Figure 9B,C).

##### Lipase Docking Results

The binding energies of the selected three compounds from the *Azara serrata* methanolic extract showed significant variations, reflecting their different inhibitory capacities. All compounds exhibited good binding energies in our docking assays compared to the known inhibitor orlistat (−8.334 kcal/mol). Nonetheless, the experimental inhibition assays indicated that the extract possessed lower inhibitory potency (Table 4). Probably, antagonistic interactions among the different compounds present in the extract interfered. Although each compound individually showed good binding energy with the catalytic site of the lipase, in the context of the extract, they could have interfered with each other in their ability to inhibit the enzyme.

**Figure 10:**
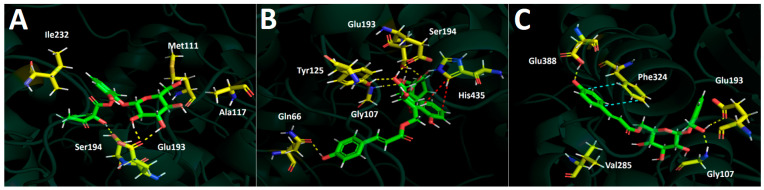
Predicted intermolecular interactions of selected compounds in the *A. serrata* methanolic extract and the residues of the human bile salt activated lipase catalytic site. Yellow dotted lines indicate hydrogen bond interactions, cyan dotted lines represent π-π interactions, and red lines represent the π-cation. (**A**). Idescarpin in the catalytic site. (**B**). Coumaroyl idescarpin in the catalytic site. (**C**). Coumaroyl idesin in the catalytic site.

Each compound separately showed the following interactions depicted in Figure 10A–C: Idescarpin exhibited four hydrogen bond interactions with Tyr125, Glu193, and Ser194. Coumaroyl idescarpin showed six different hydrogen bonds with Gln66, Gly107, Tyr125, and Glu193 and a π-cation interaction, illustrated by the red lines in figure LL-B. Finally, coumaroyl idesin formed only three hydrogen bond interactions with Gly107, Glu193, and Glu388 as well as a π-π interaction with the residue of Phe324 in the catalytic site.

##### Glucosidase Docking Results

**Figure 11:**
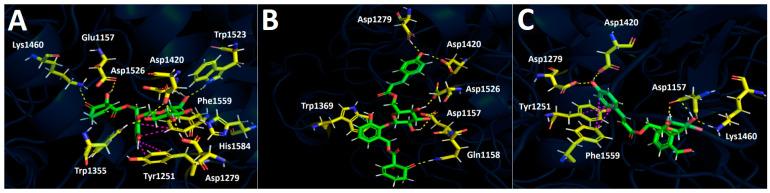
Predicted intermolecular interactions of selected compounds in the *A. serrata* methanolic extract and the residues of the human glucosidase catalytic site. Yellow dotted lines indicate hydrogen bond interactions, cyan dotted lines represent π-π interactions, and magenta dotted lines represent T-shaped interactions. (**A**). Idescarpin in the catalytic site. (**B**). Coumaroyl idescarpin in the catalytic site. (**C**). Coumaroyl idesin in the catalytic site.

Table 4 shows that the potency of glucosidase enzyme inhibition by the methanolic extract was lower compared to the known inhibitor acarbose (371.6 ± 0.0 μg/mL and 1.72 ± 0.0 μg/mL, respectively). These data are consistent with docking experiments conducted for the three selected compounds from the methanolic extract, which showed less favorable binding energies for idescarpin, coumaroyl idescarpin, and coumaroyl idesin (Table 5). This suggests that the compounds would act as glucosidase inhibitors but with slightly lower potency. Nonetheless, each compound exhibited a good interaction profile, and, therefore, each one could behave as an effective glucosidase inhibitor on its own. In this sense, idescarpin formed nine hydrogen bond interactions with Asp1157, Asp1276, Asp1420, Lys1460, Trp1355, Trp1523, Asp1526, and His1584 (Figure 11A). Furthermore, idescarpin formed two T-shaped interactions with Phe1559 and Tyr1251. Figure 11B shows that coumaroyl idescarpin executed six hydrogen bond interactions with Asp1157, Gln1158, Asp1279, Asp1420, and Asp1526. Finally, coumaroyl idesin to the glucosidase catalytic site formed hydrogen bonds with Asp1157, Asp1279, Lys1460, and Asp1420. Tyr1251 and Phe1559 formed two T-shaped interactions with the benzene ring of the p-coumaric acid framework of this derivative.

##### Amylase Docking Results

The methanolic extract showed a strong capacity to inhibit the amylase. Table 4 shows that the extract exhibited a potency comparable to that of the inhibitor acarbose, turning it into a good agent as a glucosidase inhibitor. Similarly, docking assays showed that the binding energy profiles for the selected tested compounds were very similar to each other and comparable to the energy obtained by acarbose (−12.626 kcal/mol, Table 5).

**Figure 12:**
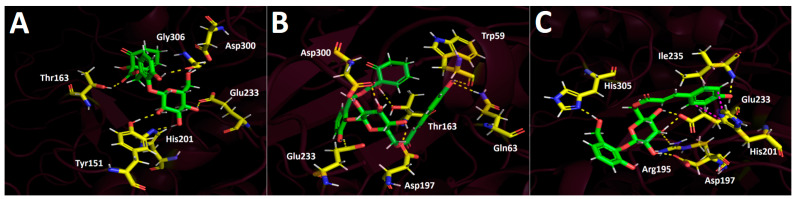
Predicted intermolecular interactions of selected compounds in the *A. serrata* methanolic extract and the residues of the human pancreatic alpha-amylase catalytic site. Yellow dotted lines indicate hydrogen bond interactions and magenta dotted lines represent T-shaped interactions. (**A**). Idescarpin in the catalytic site. (**B**). Coumaroyl idescarpin in the catalytic site. (**C**). Coumaroyl idesin in the catalytic site.

Just like in the other enzyme docking experiments, the main interactions for all derivatives were hydrogen bonds. Therefore, idescarpin formed six hydrogen bond interactions with Tyr151, Thr163, His201, Glu233, Asp300, and Gly306 (Figure 12A). Coumaroyl idescarpin executed seven hydrogen bonds with Trp59, Gln63, Thr163, Asp197, Glu233, and Asp300 (Figure 12B). Coumaroyl idesin formed seven hydrogen bond interactions with Arg195, Asp197, Glu233, Ile235, and His305, and a T-shaped interaction was also observed, which suggested a strong and stable binding configuration.

## 3. Materials and Methods

### 3.1. Plant Material

The berries of *Azara serrata* Ruiz & Pav. were collected in Valdivia’s Botanical Garden (39°48′16″ S 73°15′01″ W/−39.8045, −73.2502), southern Chile, in May 2023 and were identified and deposited with the voucher specimen UAChAS-230412. The berries were freeze-dried (Labconco Freezone 4.5 l, Kansas, MO, USA), and part of them was sealed in a vacuum bag and brought to Germany to perform the chromatographic analyses and prepare an enriched anthocyanin extract.

### 3.2. Chemicals

For the preparation of the enriched anthocyanin extract, Amberlite™ XAD-7HP was purchased from Sigma-Aldrich Co. (St. Louis, MO, USA) and Sartobind^®^ S strong acidic cation exchanger MA 75 was obtained from Sartorius Stedim Biotech GmBH (Göttingen, Germany). Chemicals used for the extraction procedure were double-deionized water (Nanopure^®^, Werner GmbH, Leverkusen, Germany), hexane (HPLC-grade), methanol (HPLC grade), and dichloromethane (HPLC grade), purchased from Fisher Scientific (Loughborough, U.K.); formic acid (ACS Reagent 99–100% purity) and ethyl acetate (HPLC grade), purchased from VWR Chemicals (Radnow, PA, USA); and sodium chloride (≥99.5%. p.a., ACS, ISO), disodium hydrogen phosphate dihydrate (≥99.0%, p.a.), and citric acid (≥99.5%, p.a.); obtained from Carl Roth GmbH & Co. KG (Karlsruhe, Germany). For the UHPLC-DAD analyses, water and formic acid were obtained as mentioned above and acetonitrile (HPLC-grade) was purchased from Honeywell Specialty Chemicals (Seelze, Germany). The solvents used for the UHPLC-TIMS-TOF analyses were water (LC-MS grade) and acetonitrile (UHPLC-MS-grade), purchased from TH. Geyer GmbH & Co. KG (Renningen, Germany), and formic acid (LC-MS grade), purchased from Fisher Scientific (Loughborough, UK). For quantitation of the anthocyanins, cyanidin-3-*O*-β-d-glucoside chloride (purity ≥ 98.00%) was purchased from Phytolab (Vestenbergsgreuth, Germany). Commercial Folin–Ciocalteu reagent, ferric chloride hexahydrate, 2,2-diphenyl-1-picrylhydrazyl (DPPH), 2,4,6-tris(2-pyridyl)-s-triazine, Trolox, dimethyl sulfoxide (DMSO), tyrosinase (EC1.14.18.1), acetylcholinesterase (*Tc*AChE, EC 3.1.1.7), butyrylcholinesterase (*h*BuChE, EC 3.1.1.8), porcine pancreatic lipase, 4-nitrophenyl-dodecanoate, phosphate buffer, dinitrosalicylic acid, L-DOPA, kojic acid, trichloroacetic acid (Merck, Darmstadt, Germany), fetal calf serum (FCS, Gibco, Grand Island, NY, USA), L-glutamine (Merck, Darmstadt, Germany), α-amylase, α-glucosidase, standard p-nitrophenyl-d-glucopyranoside, acarbose, orlistat, sodium persulfate sodium carbonate, ferrous sulfate, sodium acetate, sodium sulfate anhydrous, and absolute ethanol were obtained from Sigma Aldrich Chem. Co. (Sigma, St. Louis, MO, USA).

### 3.3. Sample Preparation

#### 3.3.1. Extract for Elucidation of the Phenolic Profiles

The extract for elucidation of the phenolic profiles was prepared by macerating approximately 30 mg of dried pulverized berries in 20 mL of a mixture of methanol/water/formic acid (49.5/49.5/1; *v/v/v*) for 12 h while stirring at room temperature. After filtration, the methanol was removed using a rotary evaporator at 200 mbar and 40 °C. The aqueous solution then was freeze-dried and then diluted in 1 mL of water/acetonitrile/formic acid (95/4/1; *v/v/v*), filtered through a 0.2 µm PTFE syringe filter, and stored at 4 °C until measured using the UHPLC-DAD-TIMS-TOF method.

#### 3.3.2. Enriched Anthocyanin Extract for Quantitation

For the quantitation of the major anthocyanins, approximately 1 g of berries was milled using a laboratory mill (IKA-Werke, Staufen im Breisgau, Germany) and then degreased using 20 mL of n-hexane; this was repeated three times. The n-hexane was discarded and the degreased berries were then extracted using the method including analytical membrane chromatography, as described by Hopfstock et al. (2024) [46]. This procedure was carried out three times. After enrichment of the anthocyanins, the extracts were diluted in 1 mL of water/acetonitrile/formic acid (95/4/1; *v/v/v*), filtered through a 0.2 µm PTFE syringe filter, and stored at 4 °C until measured by UHPLC-DAD and UHPLC-DAD-TIMS-TOF. Calibration at seven levels was prepared using cyanidin-3-*O*-β-d-glucoside. The concentrations were 75, 50, 25, 10, 5, 2.5, and 1 mg L^−1^, with each level measured in duplicate. The concentration of the anthocyanins was reported as micrograms of cyanidin-3-*O*-β-d-glucoside equivalents per gram of dry weight (DW).

#### 3.3.3. Purified Anthocyanin Extract

Approximately 10 g of dried berries was treated as described in the method above. The volume of the used solvents was upscaled to account for the higher amount of plant material. The extraction resulted in a yield of 8.9 mg of enriched anthocyanin extract, which was stored at 4 °C until being shipped to Chile for the enzyme tests.

### 3.4. Experimental Parameters

#### 3.4.1. Ultra High Performance Liquid Chromatography (UHPLC) Diode Array Detector (DAD) Analysis for the Quantitation of the Anthocyanins

For the quantitation of the anthocyanins, an Agilent 1290 Infinity II System (Agilent Technologies, Waldbronn, Germany) equipped with a binary solvent manager, an autosampler, a column heater, and a diode array detector was used. The column was a C18 Kinetex core-shell column (2.1 mm i.d. × 100 mm, 1.7 µm). The mobile phases were (A) water/formic acid (95/5; *v/v*) and (B) acetonitrile/formic acid (95/5; *v/v*), with a column temperature of 40 °C, flow of 0.3 mL min^−1^, and an injection volume of 5 µL. The gradient was as follows: at initial conditions, 3% B; at 20 min, 20% B; at 30 min, 35% B; at 35 min, 95% B; at 37 min, 95% B; at 38 min, 3% B, until 40 min. The operating software for the system was OpenLAB Version 3.4 (Agilent Technologies, Waldbronn, Germany).

#### 3.4.2. UHPLC Trapped Ion Mobility Spectrometry (TIMS) Time of Flight (TOF) Mass Spectrometry

The chromatographic analysis was performed on an Agilent 1290 Infinity system including the same parts as the system used for the qualification. The column used was an Agilent Eclipse Plus C18 RRHD (2.1 mm i.d. × 100 mm, 1.8 µm). The used mobile phases were (A) water containing 0.1% formic acid and (B) acetonitrile containing 0.1% formic acid, with a temperature of 30 °C, flow of 0.2 mL min^−1^, and injection volume of 2 µL. The gradient was as follows: at initial conditions, 2% B; at 25 min, 20% B; at 35 min, 35% B; at 40 min, 98% B; at 43 min, 98% B; at 45 min, 2% B, until 50 min. For the mass spectrometry, the system was a timsTOF equipped with an electrospray ionization source (Bruker Daltonik, Bremen, Germany). For ESI-negative measurements, the settings were *m*/*z* 20–1300; inversed ion mobility range 1/k0, 0.45–1.45 V s cm^−2^; ramp time, 100 ms; capillary voltage, 3600 V; nebulizing gas pressure, 2.20 bar (N2); dry gas flow rate, 10 L min^−1^ (N_2_); nebulizer temperature, 220 °C; collision energy, 10–50 eV (stepping). For ESI-positive measurements, the settings were scan range, *m*/*z* 100–1350; inversed ion mobility range 1/k0, 0.55–1.90 V s cm^−2^; ramp time, 100 ms; capillary voltage, 4500 V; nebulizing gas pressure, 2.20 bar (N_2_); dry gas flow rate, 10 L min^−1^ (N_2_); nebulizer temperature, 220 °C; collision energy, 10–30 eV (stepping). To calibrate the mass spectrometer and trapped ion mobility, the ESI-L Low Concentration Tuning Mix (Agilent Technologies, Waldbronn, Germany) was used; for the negative mode, the mixture was diluted by a factor of twenty. To operate the system, the software was Bruker Compass Hystar Version 6.2 and otofControl Version 6.2 (Bruker Daltonik, Bremen, Germany). For evaluating the analyses, Bruker Compass Data Analysis Version 5.3 (Bruker Daltonik, Bremen, Germany) was used.

### 3.5. Phytochemical Analysis

#### Determination of Total Phenol and Flavonoid Contents

The total phenol content (TPC) was determined using the Folin–Ciocalteau method. TPC quantification was performed based on the gallic acid standard curve and the results were expressed in μg gallic acid equivalent GAE/g (μg GAE)/g dry weight. The total flavonoid content (TFC) was determined by the aluminum chloride colorimetric method. TFC quantification was performed based on the quercetin standard curve and the results were expressed in mg quercetin equivalent (QE)/g dry weight. The total anthocyanins content (TAC) was estimated using the pH differential method [11]. The *Azara* methanolic extract was diluted with pH 1.0 and pH 4.5 buffers. The absorbance was measured at 510 nm and 700 nm for each of the buffers. TAC (expressed as cyanidin-3-glucoside) was calculated using the following equations: TAC = (A × MW × DF × V × 1000)/(ε × 1 × M) and A = (A510 − A700) pH 1.0 − (A510 − A700) pH 4.5, where DF is the dilution factor, MW is the molecular weight of cyanidin-3-glucoside (449 g/mol), V is the volume of extract, ε is the molar extinction coefficient of cyanidin-3-glucoside (26,900), and M is the mass of the *Azara serrata* extract.

### 3.6. Antioxidant Activity Assays

#### 3.6.1. Ferric-Reducing Antioxidant Power (FRAP)

The ferric-reducing antioxidant power of the samples was determined according to previous procedures [47]. Briefly, 12 mM sodium acetate trihydrate buffer, pH 3.6; 10 mM TPTZ solution (2,4,6-tripyridyl-s-triazine), made up to volume with diluted HCl (40 mM) and prepared fresh the same day as used; and 20 mM FeCl_3_·6H_2_O solution were used in the assay. FRAP reagent was prepared by mixing 1020 µL pH 3.6 buffer solution, 100 µL 10 mM TPTZ, and 100 µL 20 mM FeCl_3_·6H_2_O. The microplate reaction was performed with 10 µL sample (or standard) plus 290 µL FRAP reagent, reacted for 60 min, and then read at 593 nm. Data were expressed as µmol the Trolox equivalent (TE)/g DW.

#### 3.6.2. Free Radical Scavenging (DPPH)

Briefly, 50 µL standard or sample dissolved in EtOH plus 150 µL of DPPH solution (156 µM in EtOH) was incubated for 30 min in the dark at 35 °C. Then, the absorbance was measured at 517 nm [47]. The Trolox calibration curve was recorded between 20 and 200 µM. The results were expressed as micromoles of Trolox equivalents per g dry sample (TE/g DW).

#### 3.6.3. ABTS Assay

The ABTS assay was performed by bleaching the cationic radical ABTS•+ as described previously [47]. Briefly, ABTS stock solution was prepared by mixing ABTS and sodium persulfate in distilled H_2_O, considering a final concentration of 7 mM ABTS and a final concentration of 3.6 mM for sodium persulfate (Na_2_S_2_O_8_). The results were expressed as micromoles of Trolox equivalents per g dry sample (TE/g DW). The Trolox curve ranged between 20 and 200 µM.

#### 3.6.4. ORAC Assay

The ORAC assays were performed as previously described [47]. The reaction was prepared with 175 µL 108 nM fluorescein, 45 μL sample/Trolox/blank incubated at 37 °C for 30 min, and 50 μL AAPH (2,2′-Azobis(2-amidinopropane) dihydro-chloride). The mixture was then read for 2 h every 2 min at an excitation wave of 480 nm and an emission wave of 520 nm. The curve went from 1 to 20 µM. The results were expressed as micromoles of Trolox equivalents per g dry sample (TE/g DW).

### 3.7. Inhibition of Alpha-Amylase, Alpha-Glucosidase, and Lipase Enzymes

The ability of *Azara serrata* to inhibit the alpha-glucosidase was measured using the method described by Nampoothiri et al. [48] and then slightly modified [49]. Briefly, 50 μL 5 mM p-nitrophenyl-D-glucopyranoside and 50 μL 100 mM extract in sodium phosphate buffer (pH 6.9) were put together in a 96-well microplate and incubated at 37 °C for 5 min. Then, 0.1 U/mL glucosidase was added to each well at 37 °C and readings were performed at 405 nm for 30 min. The inhibitory effects of the extracts were expressed as IC_50_-values, which refer to the concentration that inhibits 50% of the enzyme activity. The inhibition of alpha-amylase was performed using the method described in a previous work of our group [49]. A solution of 1% starch and 100 μL extract in 20 mM sodium phosphate buffer was mixed (pH 6.9 with 6 mM sodium chloride) at 25 °C. Then, 100 μL porcine pancreatic alpha-amylase (0.5 mg/mL) was added and incubated at 25 °C for 10 min. The reaction was stopped by adding 200 μL dinitrosalicylic acid reagent at 100 °C for 5 min. The samples were cooled and 50 μL was taken from each tube and diluted by adding 200 μL water, and the absorbance was measured at 540 nm. The inhibitory effects of the extracts were expressed as IC_50_. Both activities were calculated using the following equation: Inhibition (%) = (1 − extract absorbance/control absorbance) × 100. Acarbose was included as a positive control.

The lipase assay was performed as reported [7,50]. Porcine pancreatic lipase type II was suspended in pure water at 20 μg/mL. Briefly, the assay mixture was Tris-HCl buffer (0.1 M, pH 8.5), lipase (10 mg/mL), and solutions of the extracts at different concentrations; the substrate solution (5 mM) was incubated for 15 min at 37 °C and the absorbance was measured at 410 nm on a microplate reader (BioTek Instrument, Inc., Winooski, VT, USA). The results were expressed as IC_50_-values. Orlistat was used as a positive control.

### 3.8. Inhibition of Acetylcholinesterase, Butyrylcholinesterase, and Tyrosinase Enzymes

The inhibitory activity of the ChE enzymes was evaluated as described by Ellman et al. [51]. Briefly, a solution was made with 5-dithio-bis(2-nitrobenzoic) acid (DTNB) in Tris-HCl buffer (pH 8.0) containing MgCl_2_ 0.02 M and NaCl 0.1 M. Then, the *Azara* methanolic extract (50 mL, 2 mg/mL) was mixed in a 96-well microplate with 125 mL DTNB, acetylcholinesterase (*Tc*AChE), or butyrylcholinesterase (*h*BuChE) solution (25 mL); dissolved in Tris-HCl buffer (pH 8.0); and incubated for 15 min at 25 °C. The reaction was started by the addition of acetylthiocholine iodide (ATCI) or butyrylthiocholine chloride (BTCl) (25 mL). After 10 min of reaction, the absorbance was measured at a wavelength of 405 nm and the IC_50_ (μg/mL) was calculated [49]. Tyrosinase inhibitory activity was measured using the modified dopachrome method with L-DOPA as a substrate [52]. The extract solution (25 μL, 2 mg/mL) was mixed with tyrosinase solution (40 μL) and phosphate buffer (100 μL, pH 6.8) in a 96-well microplate reader (BioTek Instrument, Inc., Winooski, VT, USA) and incubated for 15 min at 25 °C. The reaction was then initiated with the addition of L-DOPA (40 μL). The sample and blank absorbances were recorded at 492 nm after incubation for 10 min at 25 °C. The absorbance of the blank was subtracted from that of the sample and the tyrosinase inhibitory activity was expressed as the IC_50_ values, with kojic acid used as a positive control.

### 3.9. Docking Simulations

The partial charges plus the geometries of all compounds shown in Figure 6 were fully set using the DFT method, with set B3LYP-6-311G+ (d p) as the standard basis [53,54] in Gaussian 09W software (Gaussian, Inc.: Wallingford, CT, USA) [55]. Then, deprotonations and energetic minimizations were acquired using the LigPrep tool in Maestro Schrödinger suite v.11.8 (Schrödinger, LLC, New York, NY, USA) [56]. *Torpedo Californica* acetylcholinesterase structure (*Tc*AChE; PDBID: 1DX6 code [57]) plus human butyrylcholinesterase (*h*BuChE; PDBID: 4BDS code [58]) and the tyrosinase obtained from *Agaricus bisporus* mushroom (tyrosinase; PDBID: 2Y9X code [59]) were obtained from the Protein Data Bank RCSB PDB [60]. Human bile salt-activated lipase (lipase; PDBID: 1F6W code [61]), human Maltase-Glucoamylase (Glucosidase; PDBID: 3TOP code [62]), human pancreatic alpha-amylase (Amylase; PDBID: 1B2Y code [63]), and lipase according to Terzyan et al. [61] and amylase and glucosidase according to Nahoum et al. [63] and Ren et al. [62] were obtained from the Protein Data Bank RCSB-PDB [64]. Enzyme optimizations were obtained using the Protein Preparation Wizard from Maestro software, where water molecules and ligands of the crystallographic protein active sites were removed. In the same way, all polar hydrogen atoms at pH 7.4 were added. Appropriate ionization states for acid and basic amino acid residues were considered. The OPLS3e force field was employed to minimize protein energy. The enclosing box size was set to a cube with sides of 26 Å in length. The presumed catalytic sites of each enzyme in the centroid of selected residues were chosen, considering their accepted catalytic amino acids: Ser200 for acetylcholinesterase (*Tc*AChE) [65,66], Ser198 for butyrylcholinesterase (*h*BuChE) [67,68], and His263 for tyrosinase [59,69,70]**.** The Glide Induced Fit Docking protocol was employed for the final pairings [71]. Compounds were identified by the Glide scoring function in the extra-precision mode (Glide XP; Schrödinger, LLC, New York, NY, USA) [72] and selected by the best scores and best RMS values (cutting criterion: less than 1 unit) to obtain the potential intermolecular interactions between the enzymes and compounds plus the binding mode and docking descriptors. The different complexes were shown using a Visual Molecular Dynamics program (VMD 1.8.3 and Pymol 3.0 (Delano Scientific: San Carlos, CA, USA) [73].

### 3.10. Statistical Analyses

All the experiments were performed at least three times. The results were expressed as the mean plus standard deviation (SD) using the software GraphPad Prism 8. Results were compared using one-way analysis of variance (ANOVA), followed by Tukey’s HSD (honest significant difference) test (*p* < 0.05).

### 3.11. Metabolite Identification

Identification was performed (Bruker Compass DataAnalysis Version 5.3 and MetaboScape^®^ 2022, both by Bruker Daltonik, Bremen, Germany) using the exact molecular formula and MS/MS spectra and confirmed by the available literature. The MS/MS databases used were mzCloud (https://www.mzcloud.org/, accessed on 26 July 2024, Thermo Fisher Scientific), Mass Bank of North America (MoNA, http://mona.fiehnlab.ucdavis.edu, accessed on 26 July 2024), Global Natural Product Social Molecular Networking (GNPS, http://gnps.ucsd.edu, accessed on 26 July 2024), and Human Metabolome Database (HMBD, http://www.hmdb.ca/, accessed on 26 July 2024).

## 4. Conclusions

In this study, the berries of the endemic species *Azara serrata* Ruiz & Pav. from the Chilean Valdivian Forest were studied regarding their chemical composition and bioactivities. The fruits proved to be a good source of interesting phytochemicals. Apart from containing the phenolic compounds typically associated with plants from the Salicaceae family, we were able to identify a potentially novel species of anthocyanins. Further studies are required to isolate and characterize the newly detected compounds in this species.

The phenolic and anthocyanin profiles were established using UHPLC coupled with UV/Vis detection and trapped ion mobility mass spectrometry (UHPLC-DAD-TIMS-TOF). Quantification of the anthocyanin derivatives was performed using external calibration as cyanidin-3-β-d-glucoside equivalents and various spectrophotometric assays also accounted for the other phenolics. Enzyme docking calculations were performed for a selection of compounds, showing promising results regarding their activity.

Therefore, we conclude that the berries of this ancient Mapuche medicine could be used as a source of health-promoting compounds for the preparation of nutritional supplements.

## Figures and Tables

**Table 1:** Phenolic compounds and anthocyanins of the methanolic and purified anthocyanin extract of the berries of *Azara serrata* Ruiz & Pav., tentatively identified (cf. Section 3.11) by UHPLC-DAD-TIMS-TOF. pyr = pyrano-type compounds (cf. Figure 4); ac = acetylated; cou = coumaroylated. Anthocyanin species of pyrano-type compounds are shown in brackets.

Peak Number	Retention Time [min]	Compound Name	UV/Vis Max. [nm]	Molecular Formula	Theoretical Mass [*m/z*]	Detected Mass [*m/z*]	Mass Error [ppm]	Fragment Ion MS/MS [*m/z*]	CCS [Å^2^]
1	2.20	unknown		C_12_H_16_O_8_	287.0772	287.0769	1.3	125, 83	169.4
2	4.20	caffeoyl glucaric acid	212–324	C_15_H_16_O_11_	371.0620	371.0616	1.0	209, 191	185.3
3	4.82	unknown		C_16_H_19_NO_9_	368.0987	368.0981	1.7	160, 119	174.9
4	5.01	gallic acid glucoside isomer		C_13_H_16_O_10_	331.0671	331.0668	0.8	285, 123	168.3
5	5.68	caffeoyl glucaric acid isomer	198–278	C_15_H_16_O_11_	371.0620	371.0614	1.5	209, 191	171.6
6	6.28	pyrocatechuic acid glucoside	216–284	C_13_H_16_O_9_	315.0722	315.0719	0.9	153	171.6
7	6.69	gallic acid glucoside isomer		C_13_H_16_O_10_	331.0671	331.0663	2.2	313, 169	169.3
8	6.93	idesin/salirepin isomer	196–276	C_13_H_18_O_8_	301.0929	301.0928	0.5	139	165.6
9	7.37	unknown		C_16_H_14_O_11_	381.0463	381.0476	−3.5	259, 241, 139	175.5
10	7.52	unknown		C_18_H_24_O_12_	431.1187	431.1195	1.9	137, 93	187.5
11	7.80	idesin/salirepin isomer		C_13_H_18_O_8_	301.0929	301.0929	0.1	121, 59	164.3
12	8.06	pyrocatechuic acid glucoside		C_13_H_16_O_9_	315.0722	315.0719	0.8	153, 109	166.1
13	8.54	unknown		C_14_H_20_O_9_	331.1035	331.1031	1.0	285, 123	173.0
14	8.71	caffeoyl glucaric acid isomer	218–324	C_15_H_16_O_11_	371.0620	371.0617	0.8	209, 191	174.2
15	8.97	chlorogenic acid isomer	216–324	C_16_H_18_O_9_	353.0878	353.0878	1.5	191, 135	170.9
16	9.36	unknown		C_18_H_24_O_13_	447.1144	447.1140	1.0	152, 108	190.1
17	9.74	caffeoyl threonic acid isomer	216–326	C_13_H_14_O_8_	297.0616	297.0615	0.2	179, 135	159.3
18	9.85	arbutin	194–272	C_12_H_16_O_7_	271.0823	271.0823	0.3	109	156.5
19	10.07	idesin/salirepin isomer	196–274	C_13_H_18_O_8_	301.0929	301.0928	0.2	139, 121	162.6
20	11.18	caffeoyl threonic acid isomer	218–328	C_13_H_14_O_8_	297.0616	297.0615	0.5	179, 135	159.3
21	11.62	coumaroylquinic acid		C_16_H_18_O_8_	337.0929	337.0925	1.3	163, 119	179.3
22	12.31	caffeic acid glucoside		C_15_H_18_O_9_	341.0878	341.0877	0.2	179, 135	177.8
23	12.78	chlorogenic acid isomer	216–326	C_16_H_18_O_9_	353.0878	353.0874	1.0	191, 127	184.4
24	13.67	feruloylquinic acid isomer	218–324	C_17_H_20_O_9_	367.1035	367.1030	1.3	191, 134, 117	179.1
25	14.04	chlorogenic acid isomer	216–326	C_16_H_18_O_9_	353.0878	353.0877	0.2	191, 135,	184.4
26	15.96	caffeoyl threonic acid isomer	218–324	C_13_H_14_O_8_	297.0616	297.0617	−0.5	179, 135, 75	158.2
27	16.47	unknown		C_20_H_32_O_10_	431.1923	431.1917	1.3	299, 71, 59	199.9
28	16.68	unknown		C_18_H_28_O_9_	387.1661	387.1663	−0.6	369, 207, 163	186.8
29	18.62	feruloylquinic acid isomer	218–322	C_17_H_20_O_9_	367.1035	367.1032	0.7	191, 134, 93	197.2
30	19.05	verbasoside		C_20_H_30_O_12_	461.1664	461.1670	−1.1	191, 131, 89	205.8
31	20.00	unknown		C_15_H_16_N_2_O_6_	319.0936	319.0938	−0.7	142, 130, 116	181.5
32	20.32	unknown		C_19_H_26_O_10_	413.1453	413.1445	2.0	161, 133	200.7
33	21.78	idescarpin/HCH-salirepin isomer		C_20_H_24_O_11_	439.1246	439.1249	−0.7	301, 283, 139	193.1
34	22.50	idescarpinol/HCH-salirepinol		C_20_H_26_O_11_	441.1402	441.1400	0.4	301, 283 157, 139	194.3
35	23.03	unknown	218–328	C_21_H_26_O_10_	437.1453	437.1448	1.2	161, 133	215.9
36	24.90	idescarpin/HCH-salirepin isomer	196–278	C_20_H_24_O_11_	439.1246	439.1244	0.4	301, 139	217.8
37	25.98	trichocarposide isomer	220–320	C_22_H_24_O_9_	431.1348	431.1351	−0.7	307, 145, 123	219.2
38	26.42	unknown	220–314	C_21_H_26_O_9_	421.1504	421.1500	1.0	145, 117	213.1
39	26.61	populoside	220–324	C_22_H_24_O_10_	447.1297	447.1295	0.4	323, 179, 161, 132	201.8
40	26.99	unknown	220–326	C_21_H_28_O_10_	439.1610	439.1616	−1.3	161, 133	211.5
41	27.48	trichocarposide isomer	220–312	C_22_H_24_O_9_	431.1348	431.1350	−0.5	161, 133	202.4
42	27.80	unknown	220–312	C_21_H_26_O_9_	421.1504	421.1508	−0.9	137	206.4
43	28.18	salicortin isomer	220–312	C_21_H_28_O_9_	423.1661	423.1657	0.8	145, 117	215.1
44	29.48	salicortin isomer	220–312	C_21_H_28_O_9_	423.1661	423.1654	1.5	145, 117	207.5
45	29.89	flacourtin/salireposide		C_20_H_22_O_9_	405.1191	405.1189	0.5	135, 91	202.1
46	30.18	trichocarposide isomer	220–312	C_22_H_24_O_9_	431.1348	431.1345	0.7	161, 132	214.1
47	30.80	salicortin isomer	220–312	C_21_H_28_O_9_	423.1661	423.1658	0.7	145, 117	215.4
48	31.22	idescarpin/HCH-salirepin isomer	196–276	C_20_H_24_O_11_	439.1246	439.1236	2.3	[2M − H] 879.2552, 301, 139	193.1
49	31.34	HCH-idescarpin/di-HCH-salirepin isomer		C_27_H_30_O_14_	577.1563	577.1558	0.8	439, 421, 301, 139	222.4
50	31.96	cou-idesin/salirepin		C_22_H_24_O_10_	447.1297	447.1295	0.4	301, 139	201.8
51	32.31	trichocarposide isomer	220–326	C_22_H_24_O_9_	431.1348	431.1351	−0.9	161, 133	213.8
52	32.52	cou-arbutin	220–312	C_21_H_22_O_9_	417.1191	417.1189	0.4	307, 163, 145	208.5
53	32.7	cou-salicin	220–326	C_22_H_24_O_9_	431.1348	431.1340	1.8	161, 133	211.9
54	32.96	ac-salicortin	220–314	C_23_H_30_O_10_	465.1766	465.1763	0.8	423, 405, 163, 145	220.7
55	33.62	ac-cou-idesin/salirepin isomer	220–312	C_24_H_26_O_11_	489.1402	489.1401	0.3	343, 301, 139	210.2
56	34.51	unknown coumaroyl species	222–310	C_22_H_24_O_8_	415.1398	415.1398	0.1	163, 145, 117	211.9
57	34.79	ac-cou-idesin/salirepin isomer		C_24_H_26_O_11_	489.1402	489.1402	0.0	343, 301, 139	217.5
58	35.02	unknown coumaroyl species		C_22_H_24_O_8_	415.1398	415.1395	0.7	163, 145, 117	209.8
59	35.64	idescarpin/HCH-salirepin isomer	196–276	C_20_H_24_O_11_	439.1246	439.1249	−0.7	[2M − H] 879.2552, 301, 139	193.1
60	35.85	unknown	222–310	C_23_H_26_O_8_	429.1555	429.1552	0.6	145, 117	219.6
61	36.72	unknown		C_31_H_34_O_14_	629.1876	629.1864	1.8	157	232.2
62	36.78	idesin/salirepin salicylate		C_20_H_22_O_10_	421.1140	421.1135	1.3	137, 93	193.4
63	37.44	cou-idescarpin/HCH-salirepin		C_29_H_30_O_13_	585.1614	585.1613	0.1	439, 301, 139	220.7
64	37.85	ac-cou-idescarpin/HCH-salirepin isomer	196–220	C_31_H_32_O_14_	627.1719	627.1721	−0.3	481, 343, 301	232.1
65	38.30	ac-cou-idescarpin/HCH-salirepin isomer	196–220	C_31_H_32_O_14_	627.1719	627.1711	1.3	481, 343, 301	239.9
66	38.78	diac-cou-idescarpin/HCH-salirepin	222	C_33_H_34_O_15_	669.1825	669.1824	0.2	523, 481, 343	242.8
Anthocyanins and derivates									
67	13.71	delphinidin-glucoside	522	C_21_H_21_O_12_	465.1028	465.1023	1.0	303	205.2
68	14.07	delphinidin-rutinoside	522	C_27_H_31_O_16_	611.1607	611.1608	−0.3	303	236.0
69	15.52	cyanidin-glucoside	514	C_21_H_21_O_11_	449.1078	449.1075	0.7	287	201.5
70	16.29	cyanidin-rutinoside	516	C_27_H_31_O_15_	595.1657	595.1660	−0.4	287	232.7
71	16.85	delphinidin-arabinose		C_20_H_19_O_11_	435.0922	435.0921	0.3	303	200.5
72	17.12	pelargonidin-glucoside	510	C_21_H_21_O_10_	433.1129	433.1129	0.0	271	199.1
73	18.68	cyanidin-arabinose	514	C_20_H_19_O_10_	419.0973	419.0970	0.7	287	196.9
74	20.99	pyr-(delphinidin-rutinoside)	372–514	C_47_H_51_O_26_	1031.2663	1031.2664	−0.1	723, 439, 393	291.7
75	21.48	pyr-(delphinidin-glucoside)	372–512	C_41_H_41_O_22_	885.2085	885.2084	−0.3	723, 439, 393	272.7
76	21.74	pyr-(delphinidin-arabinoside)	372–512	C_40_H_39_O_21_	855.1978	855.1974	0.5	723, 439, 393	269.2
77	21.90	pyr-ac-(delphinidin-glucoside)		C_43_H_43_O_23_	927.2190	927.2192	−0.3	765, 439, 393	279.9
78	22.71	pyr-(cyanidin-rutinoside)	356–508	C_47_H_51_O_25_	1015.2174	1015.2713	0.1	707, 423, 377	289.2
79	23.26	pyr-(cyanidin-glucoside)	354–506	C_41_H_41_O_21_	869.2137	869.2135	−0.2	707, 423, 377	269.2
80	23.50	pyr-(cyanidin-arabinoside)	354–508	C_40_H_39_O_20_	839.2029	839.2032	−0.3	707, 423, 377	266.1
81	24.38	pyr-(pelargonidin-glucoside)		C_41_H_41_O_20_	853.2186	853.2179	0.8	691, 407, 361	267.4
82	24.64	pyr-base-(cyanidin-rutinoside)		C_33_H_33_O_16_	685.1763	685.1766	−0.4	377	240.3
83	25.08	pyr-base-(cyanidin-glucoside)		C_27_H_23_O_12_	539.1184	539.1183	0.2	377	219.0
84	25.52	pyr-base-(cyanidin-arabinoside)		C_26_H_21_O_11_	509.1078	509.1071	1.5	377	214.8
85	27.18	pyr-ac-(cyanidin-glucoside)		C_43_H_43_O_22_	911.2240	911.2244	−0.4	749, 423, 377	282.4
86	27.50	pyr-diac-(cyanidin-glucoside)		C_45_H_45_O_23_	953.2346	953.2345	0.1	791, 423, 377	283.4
87	30.58	pyr-ac-cou-(delphinidin-rutinoside)	376–514	C_58_H_59_O_29_	1219.3137	1219.3132	0.4	911, 765, 439, 393	325.4
88	30.84	pyr-cou-(delphinidin-glucoside)		C_50_H_47_O_24_	1031.2452	1031.2450	0.2	869, 723, 439, 393	299.6
89	30.98	pyr-ac-cou-(cyanidin-rutinoside)		C_58_H_59_O_28_	1203.3187	1203.3186	0.1	895, 749, 423, 377	322.7
90	31.17	pyr-ac-cou-(delphinidin-glucoside)	374–514	C_52_H_49_O_25_	1073.2557	1073.2562	−0.4	911, 765, 439, 393	307.4
91	31.28	pyr-cou-(cyanidin-glucoside)		C_50_H_47_O_23_	1015.2503	1015.2500	0.2	853, 707, 423, 377	296.1
92	31.52	pyr-ac-cou-(cyanidin-glucoside)	356–510	C_52_H_49_O_24_	1057.2608	1057.2607	0.1	895, 749, 423, 377	303.9
93	31.84	pyr-ac-cou-(cyanidin-arabinose)		C_51_H_47_O_23_	1027.2503	1027.2490	1.3	895, 749, 423, 377	301.8
94	32.18	pyr-ac-cou-(delphinidin-glucoside)		C_52_H_49_O_25_	1073.2557	1073.2546	1.0	911, 765, 439, 393	308.9
95	32.70	pyr-ac-cou-(cyanidin-glucoside)	352–508	C_52_H_49_O_24_	1057.2608	1057.2596	1.2	895, 749, 423, 377	306.9
96	33.29	pyr-ac-cou-(pelargonidin-glucoside)		C_52_H_49_O_23_	1041.2659	1041.2651	0.8	879, 733, 407, 361	307.3
97	33.47	pyr-diac-cou-(delphinidin-glucoside)		C_54_H_51_O_26_	1115.2663	1115.2668	−0.4	953, 807, 439, 393	311.4
98	34.15	pyr-diac-cou-(cyanidin-glucoside)		C_54_H_51_O_25_	1099.2714	1099.2720	−0.5	937, 791, 423, 377	308.7
99	34.29	pyr-ac-cou-(delphinidin-glucoside)		C_52_H_49_O_25_	1073.2557	1073.2560	−0.3	911, 765, 439, 393	304.8
100	34.39	pyr-ac-cou-(cyanidin-glucoside)		C_52_H_49_O_24_	1057.2608	1057.2608	0.5	895, 749, 423, 377	304.2

**Table 2:** Results of the quantitative analysis of the enriched anthocyanin extract measured by UHPLC-DAD and reported as cyanidin-3-O-β-d-glucoside equivalents. Pyr = pyrano; ac = acetylated.

Peak Number	Retention Time [min]	Compound Name	µg/g DW Berries
67	5.32	delphinidin-glucoside	81.20 ± 8.00
68	6.22	delphinidin-rutinoside	20.45 ± 1.90
69	6.66	cyanidin-glucoside	115.8 ± 9.38
70	7.76	cyanidin-rutinoside	21.37 ± 1.58
71	8.13	delphinidin-arabinoside	7.59 ± 0.43
73	9.67	cyanidin-arabinoside	7.99 ± 0.35
74	11.07	pyr-delphinidin-rutinoside	14.65 ± 1.42
75	11.42	pyr-delphinidin-glucoside	37.73 ± 3.13
76	11.62	pyr-delphinidin-arabinoside	4.23 ± 0.35
78	12.81	pyr-cyanidin-rutinoside	17.37 ± 1.18
79	13.15	pyr-cyanidin-glucoside	75.55 ± 4.67
80	13.41	pyr-cyanidin-arabinoside	4.62 ± 0.20
87	20.66	pyr-ac-coumaroyl-delphinidin-rutinoside	3.21 ± 0.36
90	21.49	pyr-ac-coumaroyl-delphinidin-glucoside	9.61 ± 0.69
92	22.29	pyr-ac-coumaoryl-cyanidin-glucoside	18.66 ± 1.18
95	23.58	pyr-ac-coumaroyl-cyanidin-glucoside	1.47 ± 0.11
sum			441.49 ± 34.93

**Table 3:** ^a^ TAC expressed as μg cyanidin glucoside equivalent CGE/g dry weight; ^b^ TFC expressed as μg QE/g dry weight; ^c^ TPC expressed as μg GAE/g dry weight; ^d^ DPPH, ABTS, ORAC, and FRAP expressed as μmol TE/g dry weight.

Sample	*A. serrata* Fruits
TAC ^a^	500.40 ± 9.67
TFC ^b^	2400.64 ± 94.45
TPC ^c^	5700.410 ± 47.24
DPPH ^d^	350.301 ± 10.56
ABTS ^d^	490.626 ± 7.87
ORAC ^d^	387.21 ± 5.27
FRAP ^d^	426.96 ± 15.23

**Table 4:** Enzymatic inhibitory activity (IC50, in µg/mL) of *A. serrata* methanolic extract against *h*BuChE, *Tc*AChE, tyrosinase, lipase, glucosidase, and amylase.

Assay	*h*BuChE	*Tc*AChE	Tyrosinase	Lipase	Glucosidase	Amylase
*A. serrata* extract	12.24 ± 0.03	3.92 ± 0.23	11.12 ± 0.10	32.43 ± 0.0	371.6 ± 0.0	7.23 ± 0.0
Galantamine	3.81 ± 0.02	0.57 ± 0.03	-	-	-	-
Acarbose	-	-	-	-	7.25 ± 0.0	6.56 ± 0.0
Orlistat	-	-	-	1.72 ± 0.0	-	-
Kojic acid	-	-	0.73 ± 0.04	-	-	-

All values are expressed as IC_50_, means ± SD (*n* = 3).

**Table 5:** Binding energies obtained from docking experiments of selected compounds from the *A. serrata* methanolic extract, as well as the known inhibitors galantamine, acarbose, orlistat, and kojic acid, over acetylcholinesterase (*Tc*AChE), butyrylcholinesterase (*h*BuChE), tyrosinase, lipase, glucosidase, and amylase.

Compound	Binding Energy (kcal/mol) Acetylcholinesterase	Binding Energy (kcal/mol) Butyrylcholinesterase	Binding Energy (kcal/mol) Tyrosinase	Binding Energy (kcal/mol) Lipase	Binding Energy (kcal/mol) Glucosidase	Binding Energy (kcal/mol) Amylase
**Idescarpin**	−17.847	−15.512	−11.054	−12.127	−12.160	−9.210
**Coumaroyl idescarpin**	−16.071	−15.115	−10.050	−12.831	−11.933	−10.150
**Coumaroyl idesin**	−18.210	−12.103	−10.009	−12.605	−11.032	−11.074
**Galantamine**	−12.989	−7.125	----	----	----	----
**Acarbose**	----	----	----	----	−18.591	−12.626
**Orlistat**	----	----	----	−8.334		----
**Kojic acid**	----	----	−6.050	----	----	----

## Data Availability

The data that support the findings of this study are available from the corresponding authors upon reasonable request.

## Supplementary Materials

The following supporting information can be downloaded at https://www.mdpi.com/article/10.3390/plants13192756/s1: Figure S1: *UHPLC-DAD-TIMS-TOF* analysis of the purified anthocyanin extract of the berries from *Azara serrata* Ruiz & Pav.: base peak all MS positive **I**, DAD signal at 280 nm **II**, and DAD signal at 520 nm **III**.; Figure S2: Chromatogram of the UHPLC-DAD analysis of the purified anthocyanin extract of the berries of *Azara serrata* Ruiz & Pav. at 280 nm.; Figure S3: External calibration curve of cyanidin-3-O-β-d-glucoside ranging from 1 to 75 mg L^−1^.
