# Supplementary material for: In Vitro Inhibition of Enzymes and Antioxidant and Chemical Fingerprinting Characteristics of Azara serrata Ruiz & Pav. Fruits, an Endemic Plant of the Valdivian Forest of Chile
Source: Plants (Basel). 2024 Sep 30;13(19):2756. doi: 10.3390/plants13192756 (PMC11478526; doi:10.3390/plants13192756)
Supplement: Supplementary file 1 [file plants-13-02756-s001.zip › plants-3209437-supplementary.pdf]

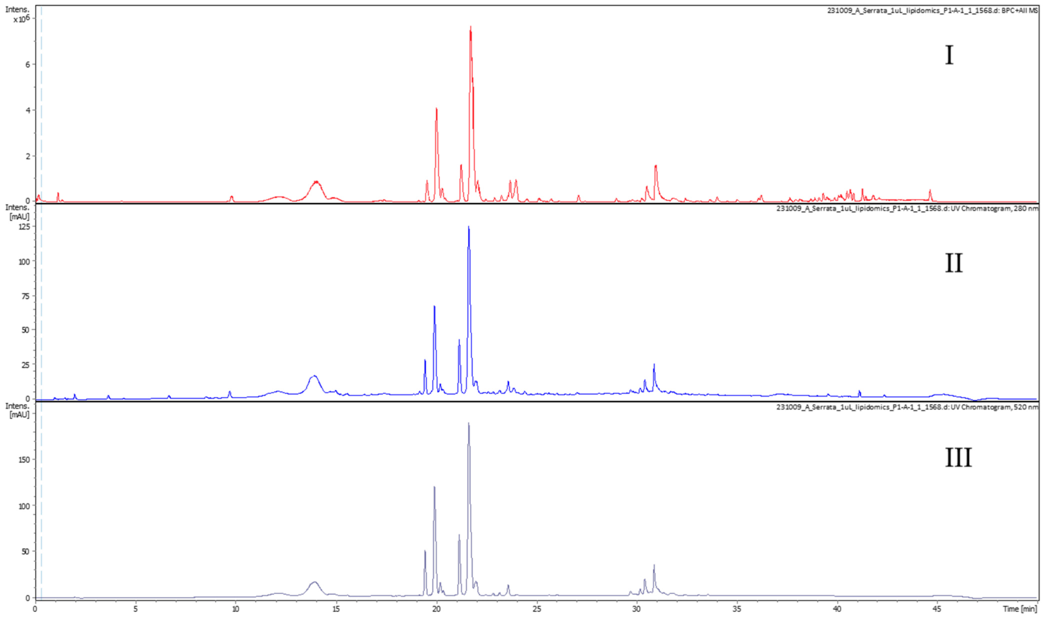


**Figure S1.** UHPLC-DAD-TIMS-TOF analysis of the purified anthocyanin extract of the berries from *Azara serrata* Ruiz & Pav. With **I** Base Peak All MS positive **I**, DAD signal at 280 nm **II**, DAD signal at 520 nm **III**.

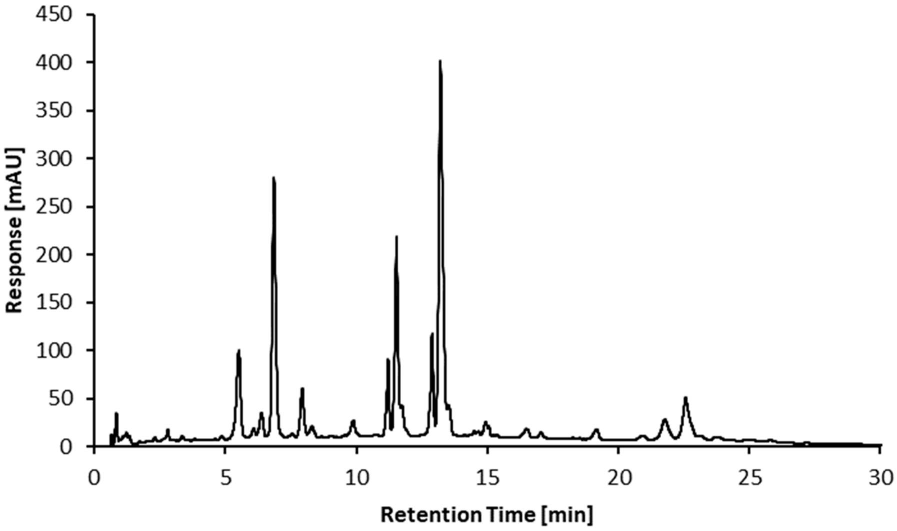


**Figure S2.** Chromatogram of the UHPLC-DAD analysis of the anthocyanin rich extract of the berries of *Azara serrata* Ruiz & Pav. at 280 nm.

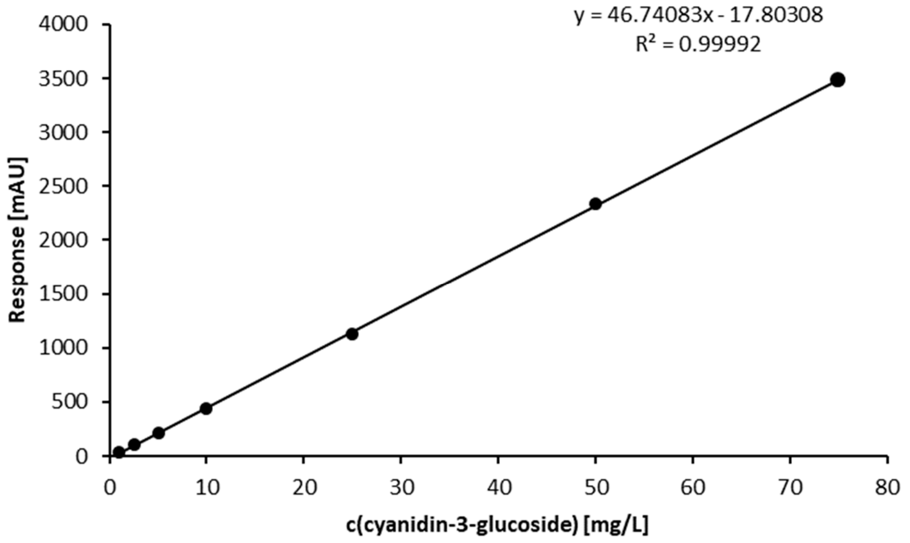


**Figure S3.** *External calibration curve of with cyanidin-3-O-β-D-glucoside ranging from 1 – 75 mg L$^{-1}$.*
